# Supplementary material for: Active TB case finding in a high burden setting; comparison of community and facility-based strategies in Lusaka, Zambia
Source: PLoS One. 2020 Sep 10;15(9):e0237931. doi: 10.1371/journal.pone.0237931 (PMC7482928; doi:10.1371/journal.pone.0237931)
Supplement: Attachment — Submitted filename: PONE-D-20-06815.pdf [file pone.0237931.s002.pdf]

PONE-D-20-06815 Reviewer

1. Please make it clear what the aim of the study is and how it can be measurable. both in the abstract and in the text
2. Describe the community, is it around urban area? Urban or rural? where are the health facilities located serving the population? What population do these health facilities serve? is it differentiated in terms of knowledge level, socio-economic status?
3. Were there differences in the time of sputum collection and sample reception? Those patients were informed about how to collect sputum correctly?

4. Please Clarify what are the criteria for rejection of sputum (sputum not evaluated 2,173).
5. Explain in the discussion probable cause of low MTB detection in the sputum (only 506).
6. In line 107 until 109 you describe different symptoms for TB including duration of cough but in table 1. You describe statistics of cough and symptoms, please clarify if you include or no the cough with other symptoms.
7. Was the information from line 107 to 109 used to describe typical characteristics of TB?
8. improve the methodology and results in the abstract. so you can answer first the objective of the study.
   A total of 18,194 individuals were screened through the facility 9,846 (54.1%) and 8,348 (45.9%) were screened through the community. 9,309 (51.2%) were male…..
