# Supplementary material for: Active TB case finding in a high burden setting; comparison of community and facility-based strategies in Lusaka, Zambia
Source: PLoS One. 2020 Sep 10;15(9):e0237931. doi: 10.1371/journal.pone.0237931 (PMC7482928; doi:10.1371/journal.pone.0237931)
Supplement: Attachment — Submitted filename: RESPONSE TO REVIEWERS.docx [file pone.0237931.s003.docx]

Dr. Mary Kagujje

Centre for Infectious Disease Research in Zambia

P.O Box 34681. Lusaka

29^th^ June, 2020

The Editor-in-Chief
PLOS
1160 Battery Street
Koshland Building East, Suite 225
San Francisco, CA 94111
United States

Dear Sir/Madam,

**RE**: **Submission of revised manuscript in response to comments from the peer reviewers**

On behalf of all the authors on the manuscript PONE-D-20-06815 entitled “Active TB case finding in a high burden setting; comparison of community and facility-based strategies in Lusaka, Zambia”, I would like to thank you so much for the positive evaluation of our work.

We are very grateful to the reviewers for taking time to critique our work and have revised the manuscript to respond to their comments. Detailed response to the reviewers’ comments is annexed to this letter.

In addition, we have included three additional authors. They played a critical role in data base design and data curation and have been involved in review and approval of the revised manuscript. I look forward to your positive consideration of the manuscript.

Yours sincerely,

M.K

Dr. Mary Kagujje

**DETAILED RESPONSE TO REVIEWERS COMMENTS**

| Reviewer | Comment | Author response |
| --- | --- | --- |
| Reviewer 1 | Active case finding for tuberculosis (TB) is recommended by the World Health Organization as an approach to find particularly in high-burden settings, that why that manuscript is so relevant for the control of TB in development world. | Thank you |
|  | Line 107-History of cough, fever, night sweats, weight loss, chest pain and loss of appetite was documented for all patients presenting for TB screening; The authors should clarify if all the TB symptoms screening were used based WHO's recommended | We have clarified that cough, fever, weight loss and night sweats are the WHO recommended symptoms for TB screening and that loss of appetite and chest pain are recommended by the Zambia TB guidelines.  See methods section, line 127-130, Page 7 |
|  | The authors should explain how the diagnostic testing followed the standard of care for TB diagnosis in Lusaka. How were all sputum samples dropped off in study clinics and transported to the laboratory or clinic that have a facility like Xpert MTB/RIF testing. | We have clarified that our algorithms are similar to the standard of care in Zambia. See methods section, line 132-134, page 7.  The project installed a geneXpert instrument at the study site, George clinic. All the samples, both from the community and the facility, were transported to the laboratory for geneXpert MTB/RIF testing on the same day of collection by community health workers. This has been clarified in the methods section, line 103, page 6 and line 142-143, page 7. |
|  | In data analysis, the authors should be expanded and clarified to ensure that readers understand exactly what the researchers studied. Alternatively, they could include another statistical method as Poisson regression to fit the different variables. So that, the tables will be easier to understand. | Thank you for the feedback  We have made various changes to the data analysis section to make it more clear. See methods section, line 168-189, page 8-9.  Additionally, Poisson regression would not be suitable statistics to run on the data as this was a prospective cross sectional study. |
| Reviewer 2 | The abstract is well written but the results reporting could benefit from clarifying the denominators of each of the proportions reported and these out to be restricted to the samples tested. Only then will it be possible to assess if the conclusions are appropriate for the results | Thank you. The denominators have been added to the parts where they were missing. See abstract section, line 41 page 2 |
|  | Line 51, the authors state that 24,929 cases are missed in Zambia, this predominantly occurs in In peri urban setting and that 50% missed at facilities. Can they expand on why 50% are missed at facilities, is it a methodological?, awareness?, capacity problem? Ultimately this is what the study aims to narrow ....as a gap!! | Thank you. We have included the various reasons why TB cases are missed. See introduction section, line 59-62, page 4 |
|  | The introduction in general lacks the context within which the study is set, how do i evaluate the intervention, who has used this interventions in the way you have designed it?, what does WHO recommend,  Zambia's TB incidence is ~ 208 per 100,000 persons per year, So, comparatively how is this approach expected to improve ACF relative to where it has been implemented?... will you approach improve time to identification, cost of extra case identified, or raw numbers of identified?... what is your measure of improvement. With such a triangulation of information, one could fully understand what you are doing, how it compares elsewhere in terms of benefits and the anticipated improvements in ACF at detection level and cost involved... | Thank you so much. The context of the study is provided under methodology section, line 87- 103, page 5-6.  We have clarified that our algorithms are WHO recommended and are similar to the standard of care Zambia. See methods section, line 132-134, page 7.  Our approach did not measure time to identification but rather measured increase in TB case detection by using a quasi-experimental approach, before and after comparison.  The overall case finding approach was measured in terms additional cases detected and change in TB notification rate. This was been clarified in the introduction section, line 81-83, page 5.  Facility case finding and community case finding are being compared in terms of yield of bacteriologically confirmed TB diagnosis. See introduction section, line 82-83, page 5. Yield has been defined as bacteriologically confirmed TB cases detected among those who submitted sputum. See methods section line 172-173, page 9.  The comment about TB incidence in Zambia is noted. In the methods section, under study setting and study population sub section, we have included the notification rates for Lusaka district as well as the pre- intervention notification for George health facility to better contextualise the ACF relation to notification rate. See methodology section, line 94- 98, page 5.  We have noted the comment of the cost of each additional case found. The costing aspect of this study will be reported in separate manuscript. |
|  | There is limited information about the study site and population. For example one can not make out the geographic distribution of the population used in your study, this is critical information as the prevalence of TB by such sub-locations is likely to be known, it helps one evaluate the appropriateness of the communities to include and the depth of the followup all of which determine how the scarce resources are apportioned | Thank you so much. Geographical distribution and location of the population has been included. See methods section, line 87-92, page 5 |
|  | What is the data availability policy to enhance the provenance of this? | The study database has been shared |
|  | The authors have not explained the statistical methods used, so it makes it hard to understand what analysis was done. In my opinion there has been limited statistical input to the work with the samples size used, a number of approaches could have been used to robustly analyse the data | Thank you for the feedback  We have made various changes to the data analysis section in order to make it more clear. See methods section, line 168-189, page 8-9. |
|  | The authors use the work Impact on case finding?, how is impact defined, raw numbers, proportion, cost per sample found?, lives potentially save from exposure | The overall case finding approach was measured in terms additional cases detected and change in TB notification rate. This was been clarified in the introduction section, 81-83, page 5.  Facility case finding and community case finding are being compared in terms of yield. See introduction section 82-83, page 5. Yield has been defined as bacteriologically confirmed TB cases detected among those who submitted sputum. See methods section line 172-173, page 9. |
|  | I see tables cited in materials and methods, to me this would mean results are being reported in MM | Thank you, the citations have been removed |
|  | It is shame, with this amount of data, alot of statistical analysis to properly unpick these characteristics There samples are selected through a number of non-random selection criteria which makes extrapolations difficult We need to be sure of the denominators used. From your selection procedure, you screened ~ 50%/50% the community and hospital and the positive cases 91.5% and 8.5%, could you give an idea of what it costs to identify these (0.08*8,348).... actually the denominator should be the the samples tested. Actually it appears the prevalence is 14.3% among tested at the facility and 3.6 in the community | Thank you.  The analysis of data follows the TB diagnostic cascade. At each level of the cascade, the denominator changes. This is reflected in the flow diagram and has been clarified under the methods section, line 169-173, page 8-9.  We have added denominators to the figures in the results section, line 199-247, page 10-12  Thank you, information on cost will be provided in a different manuscript  Indeed, the yield is dependent on the samples tested. The overall yield for facility and community case finding case finding were 13.7% and 3.1% respectively. This is shown in the results section, Line 216, page 11 and line 226 page 11 respectively |
|  | What is the utility of p-values on line 187, in this case?? are you detecting more than you would expect? I suspect the best use of this would be to establish that the ACF with and without any in intervention was significantly different i.e. Q3 2016, Q3 2017... that is hwre the statistical differences would carry more weight!! | The p-values were originally intended to show that there was a statistically significant difference between the yield for community and facility case finding. To improve the flow of the results section, the content on line 185-187 which include the p-values has since been deleted and the yields put under the respective sections.  Thank you for this recommendation. However, we want to focus this data on the trend in TB notifications |
|  | On line 194 you introduce, incidence at national level, is this published or is it data that comes from the your database. How can that be independently verified, or is this peer reviewed data | The notification rate is for the facility. We have provided data on George notifications and the population in 2016, 2017 and 2018 so that it is possible to verify the notification rate. A clear table on this is provided in results section, line 255, page 13 |
|  | It would be better if table 4 showed temporal gain rather than a compounded cumulative change | We don’t understand what the reviewer is suggesting. We would like further explanation on this for us to adequately respond. |
|  | I am unsure what the proportions represent, (in reference to the query raised on the denominator), it makes it rather hard to follow the discussion | Thank you. We have added denominators to the proportions in the discussion section, line 301 page 15 |
| Reviewer 3 | The very important topic needs only a punctual review to be published. the conclusions could be further elaborated to guide health systems in Africa.the objectives must be clear, the methodology described and the discussion of results must be aligned with the results presented | Thank you so much.  We have made some few changes to the conclusion, see conclusion section line 313- 327, Page 15-16  We have clarified the objectives, see introduction section, line 78-83, Page 5  Various changes have been made to the methods and discussion section, please see respective sections.  Methods section, line 86-189, page 5-9 and discussion section, line 258-310, page 13-15 |
| PONE-D-20-06815 Reviewer | Please make it clear what the aim of the study is and how it can be measurable. both in the abstract and in the text | Thank you  This has been clarified  See abstract section, line 25-29, page 2 and introduction section, line 78-83, page 5 |
|  | Describe the community, is it around urban area? Urban or rural? where are the health facilities located serving the population? What population do these health facilities serve? is it differentiated in terms of knowledge level, socio-economic status? | Thank you.  This has been clarified under methods section, line 87-92, page 5 |
|  | Were there differences in the time of sputum collection and sample reception? Those patients were informed about how to collect sputum correctly? | We have clarified that samples were received in the laboratory on the same day that they were collected. See methods section, line 142-143, page 7  All patients were instructed on how to collect sputum samples. See methods section, line 141-142, page 7 |
|  | Please Clarify what are the criteria for rejection of sputum (sputum not evaluated 2,173). | Samples were rejected by the laboratory if: i) the specimen was leaking out into biohazard bag, ii) the sputum contained many food particles, iii) the volume was less than <0.5mls and if the sputum contained a lot of blood.  See methods section, line 143-145, page 7 |
|  | Explain in the discussion probable cause of low MTB detection in the sputum (only 506). | The 506 cases were detected from 3528 presumptive TB cases giving a yield of 14.3%. This was higher than the 10% target provided in the Zambia National TB guidelines. See discussion section, line 274-275, page 14 |
|  | In line 107 until 109 you describe different symptoms for TB including duration of cough but in table 1. You describe statistics of cough and symptoms, please clarify if you include or no the cough with other symptoms. | Cough is included in the other symptoms. Cough has now been removed since it is included in the other symptoms |
|  | Was the information from line 107 to 109 used to describe typical characteristics of TB? | We have clarified that cough, fever, weight loss and night sweats are the WHO recommended symptoms for TB screening and that loss of appetite and chest pain are recommended by the Zambia TB guidelines.  See methods section, line 127-130, Page 7 |
|  | improve the methodology and results in the abstract. so you can answer first the objective of the study. A total of 18,194 individuals were screened through the facility 9,846 (54.1%) and 8,348 (45.9%) were screened through the community. 9,309 (51.2%) were male..... | Thank you so much.  Both methodology and results section have been revised. See abstract section, line 31-44, page 2 |
